# Polymorphisms in protamine 1 and 2 genes in asthenozoospermic men: A case-control study

**Published:** 2018-06

**Authors:** Ali Nabi, Mohammad Ali Khalili, Mojgan Moshrefi, Mohammad Hasan Sheikhha, Ehsan Zare Mehrjardi, Hamid Reza Ashrafzadeh

**Affiliations:** 1 *Research and Clinical Center for Infertility, Yazd Reproductive Sciences Institute, Shahid Sadoughi University of Medical Sciences, Yazd, Iran.*; 2 *Department of Reproductive Biology, Shahid Sadoughi University of Medical Sciences, Yazd, Iran.*; 3 *Medical Biotechnology Research Center, Ashkezar Branch, Islamic Azad University, Ashkezar, Iran.*

**Keywords:** Protamine, Asthenozoospermia, Single nucleotide polymorphisms

## Abstract

**Background:**

Asthenozoospermia is one of the etiologies for male factor infertility. It was shown that any abnormality in protamines genes, reduction of protamines transcript and protamines deficiency may play a key role in asthenozoospermia.

**Objective:**

The aim of the current study was the evaluation of protamine-1 and 2 genes (*PRM1* and *PRM2*) polymorphisms in asthenozoospermic men.

**Materials and Methods:**

In this case-control study, the samples were corresponded to asthenozoospermic specimens of infertile men. The normozoospermic samples were considered as the control group. DNA sequence amplification was performed using four PRM1 and PRM2 primers, designed from 5' to 3' flank regions. The human *PRM1* and *PRM2* gene sequences were screened in search of potential mutations in highly prevalent polymorphism regions in asthenozoospermia versus normozoospermia.

**Results:**

Totally, nine highly prevalent polymorphism regions between the forward and reverse primers were screened. Three of them corresponded to *PRM1* and six to *PRM2*. The most prevalent polymorphism regions in *PRM1* were related to 102G>T (rs35576928), 49C>T (rs140477029) and 139C>A (rs737008). In the *PRM2*, 6 highly prevalent polymorphisms regions were screened, including 248C>T (rs779337774), 401G>A (rs545828790), 288C>T (rs115686767), 288G>C (rs201933708), 373C>A (rs2070923), and 298G>C (rs1646022). The allele frequencies of three upper mentioned single nucleotide polymorphisms in asthenozoospermic men including 373C>A, 298G>C and 139C>A was higher than the control group.

**Conclusion:**

Our findings indicated that the frequency of some altered genotypes in asthenozospermia was slightly higher than control group. We proposed more extensive studies to be sure that; these genotypes can precisely be related to diagnosis of asthenozoospermia, as the molecular markers.

## Introduction

In about 50% of infertile men, the underlying cause of infertility is unknown (1). Most of the male factor infertilities are as the result of poor sperm quality. Male factor infertility is a complex problem with different etiologies. Genetics, along with acquired factors, are two major factors of male infertility. It is thought that genetic abnormalities account for 15-30% of male infertility (1). In fact, accurate hormonal hemostasis and spermatogenesis are classified under the genetic factors (2). 

Although 10-15% of the sperm's chromatin is packaged by histones, the remnant is replaced by protamine. Protamine causes permanent remodeling of chromatin which leads to sperm DNA condensation. So that, during spermiogenesis, the sperm nuclear histones are exchanged by protamines which is mediated by transition proteins I and II. Then, in the late stage of spermatid elongation, triphosphopyridine nucleotide (TPN) I and II are replaced by protamine. There are two types namely: protamine I (P1 or PRM1) and protamine II (P2 or PRM2). The first one is encoded by a single-copy gene, whereas the other is related to the protamine II proteins family (P2, P3, and P4). The combination of P1 and P2 with sperm nucleic acid resulted to more chromatin condensation, solidification and very compact DNA. This chromatin compaction is required for normal sperm function, hydrodynamic shape, motility and protection of genetic information. Any changes in the protamine genes may result to changes in their expression, P1:P2 ratio and sperm condensation (3). P2 is more critical for maintaining male fertility. P1 is synthesized in the form of mature protein, but P2 is the precursor (4). 

Any changes in these genes sequences may lead to mutations or polymorphisms which would have worse effect on male fertility. For example, the outcomes may cause an undesirable influence on sperm counts like oligospermia (5), azoospermia (6, 7), and teratozoospermia (8). 

Mutation is abnormal changes of DNA sequence; while polymorphism is the existence of a gene in several allelic forms, a variation or alteration in specific DNA sequence and has been found to be very common. Research is still ongoing to ascertain the pathophysiological effects of genetic polymorphisms on male infertility. It is believed that single nucleotide polymorphisms (SNPs) can modify the function of genes and its occurrence is high in the population, with varying subtle effects. Gázquez and colleagues reported that -190C>A is related to increased protamine P1/P2 ratio and abnormal sperm morphology (9). Also, some of the SNPs associated with the male reproductive system function have an adverse effect on sperm production, motility and hormone sensitivities (10). Some alterations in protamines like protamine gene mutation, abnormal transcription regulation, expression deregulation and unsatisfactory post-translational processing can lead to male infertility. In addition, protamine mutations account for abnormal spermatogenesis which results to sperm DNA break and chromatin damages. Therefore failure in sperm penetration and embryo development happen (11).

It has been shown that protamines mutation and polymorphism occur in infertile men (10-12). More than 20 SNPs have been reported for both kinds of protamine and most of them are associated with azoospermia, subfertilities and infertilities (12). Reduced sperm motility is considered as asthenozoospermia, present in infertile men, but its etiology is unknown in the majority of cases (13). Numerous studies have reported different SNPs in protamines in relation to male infertility or azoospermia (10, 11). There are various environmental factors in diverse populations which may have influence on sperm protamine. So, different geographical regions, the limitation of experimental samples and the genetic differences between various ethnical populations are key factors which may lead to different SNPs in protamines (13). However, to the best of our knowledge, there is no study on the evaluation of protamine polymorphism in asthenozoospermia patients. Therefore, this research evaluated the polymorphism of PRM1 and PRM2 in men, diagnosed with asthenozoospermia.

## Materials and methods

**Sample collection**

A total of 179 specimens were collected from Iranian men (aged 20-40 yr) referred to Yazd Reproductive Sciences Institute, Yazd, Iran. From 2015 To 2017 in two groups: asthenozoospermic (case group, n=92) and normozoospermic (control group, n=87) men. Collection processes were the same in two groups. According to the World Health Organization guidelines, the selection was based on the sperm total motility of <32% (14). 

The control group was compromised from the normozoospermia men (total motility ˃32%), who were referred for the female infertility factor. A comprehensive andrological and physical examination was done for all participants. The exclusion criteria were as follows: varicocele, cryptorchidism, iatrogenic infertility, testis trauma, previous genital infections, exposure to chemotherapeutics or radiation, Klinefelter’s, cystic fibrosis, addiction, smoking, alcohol drinking and environmental exposure like driving job, miners, bakers and workers of chemical plants. After 3 days of abstinence, semen samples were collected in sterile containers. Following liquefaction, the samples were analyzed under the standard of the 2010 “World Health Organization” (14).

**Direct sequencing of polymerase chain reaction (PCR)-amplified products**

The gene information of PRM1 and PRM2 are as follows (NCBI database): 

PRM1: [Homo sapiens (human)], Gene ID: 5619, Gene type: protein coding, Location: 16p13.13, Exon count: 2.

PRM2: [Homo sapiens (human)], Gene ID: 5620, Gene type: protein coding, Location: 16p 13.13, Exon count 2.

Venous blood samples were collected from all participants DNA samples were extracted from total leukocyte blood using the Qiagen extraction kit (QIAamp DNA Mini Kit, USA). DNA sequence amplification was performed using four primers, designed from the 5' to 3' flank regions (Table I) on the basis of the Domenjoud and colleagues study (15). For PRM1 gene, the forward primer had 24 nucleotides and was started from nucleotide -42 to nucleotide -19 upstream of the transcription site while the reverse primer had 24 nucleotides and was started from nucleotide 492 to nucleotide 515, downstream of the polyA (AATAAA) sequence. By applying these primers, the fragment of 557 bp was amplified for PRM1. Similarly, a 599 nucleotides fragment (from -49 to -648) was amplified for the PRM2 gene.

The PCR products were amplified, using the following thermocycling program: Denaturing at 94^o^C for 5 min, 35 cycles at 94^o^C for 30 sec, annealing 70^o^C for 45 sec (P1: 70^o^C and P2: 68^o^C), extension at (P1: 72^o^C and P2: 75^o^C) for 40 sec and final hold at 72^o^C for 10 min. The termination was at 20^o^C for 2 min. The reactions were assembled using 12.5 μL master-mix, 2 μL forward and reverse primers, 1 μL DNA and 9.5 μL Nuclease-free water. Electrophoresis was carried out using 1.5% Tris-Boric acid- Ethylenediaminetetraacetic acid (EDTA) agarose gel compromising 1μg/mL ethidium bromide to ensure the intended PCR products were accurately isolated. Thereafter, identification of SNPs in the PRM1 and PRM2 genes was detected by direct sequencing of separated DNA fragments. The PCR products were purified by using standard guanidine-HCl purification. For obtaining forward and reverse sequence trace file (in ABI format) from PRM1 and PRM2 gene products, an ABI 3700 capillary DNA sequencing machine (Applied Biosystems, CA, USA) was used. 

Nine highly prevalent polymorphism regions, which were reported previously in human PRM1 and PRM2 gene sequences, were screened in search of potential mutations in men, diagnosed with asthenozoospermia compared to normal men. 

These nine regions were related to the following polymorphisms including: 102G>T (rs35576928), 49C>T (rs140477029) and 139C>A (rs737008) in PRM1 and 248C>T (rs779337774), 401G>A (rs545828790), 288C>T (rs115686767), 288G>C (rs201933708), 373C>A (rs2070923) and 298G>C (rs1646022) in PRM2. Patterns of detected polymorphisms and their frequency were compared by analysis between the asthenozoospermia and control populations. The reference sequences were obtained from gene bank (www.ncbi.nlm.nih.gov). The nucleotide bases were numbered according to their position in the genome. They were numbered from the first adenosine base of the methionine of the ATG start codon. For example, the SNP 401 G>A refers to the guanidine base which is substituted with an adenosine at position 401 from the first nucleotide of the start codon.

**Ethical consideration**

This study was approved by the Ethics Committee of Yazd Reproductive Sciences Institute, Yazd, Iran (IR.SSU.RSI.REC. 1394.14) and a written informed consent was obtained from all participants.

**Statistical analysis**

The genotypes were in H-W equilibrium and the results were analyzed by SPSS (Statistical Package for the Social Sciences, version 20.0, SPSS Inc., Chicago, Illinois, USA) software. The differences between the groups were compared using Chi-square test (p<0.05 was considered statistically significant). 

**Table I:** Forward and reverse primer sequence of *PRM1* and *PRM2*

**Gene**	**Forward primer**	**Reverse primer**
PRM I	5’-CCCCTGGCATCTATAACAGGCCGC-3’	5’-TCAAGAACAAGGAGAGAAGAGTGG-3’
PRM II	5’-AGGGCCCTGCTAGTTGTGA-3’	5’-CAGATCTTGTGGGCTTCTCG-3’

PRM: Protamine

## Results

**Figure 1:**
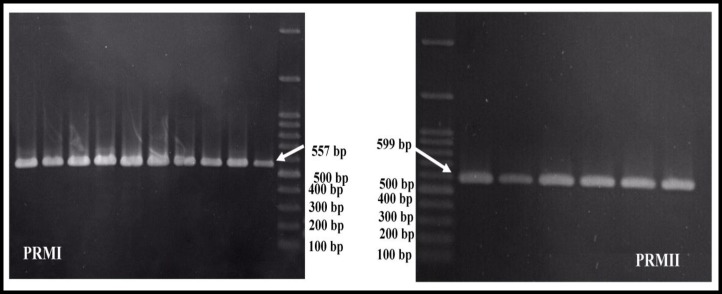
Gel electrophoresis of PCR products by designed primers for *PRM1* and *PRM2*.

The mean age of participants was 34±3.26 yr in case group and 35±2.91 yr in control. The gel electrophoresis of PCR product demonstrated that the genes of *PRM1* and *PRM2* were amplified correctly by designed forward and reverse primers. The PCR amplified 557 bp and 599 bp DNA fragments for *PRM1* and *PRM2* genes, respectively (Figure 1). Then, the allele change frequency was compared in asthenozoospermic men with control using DNA sequencing of amplified fragments.

**Figure 2:**
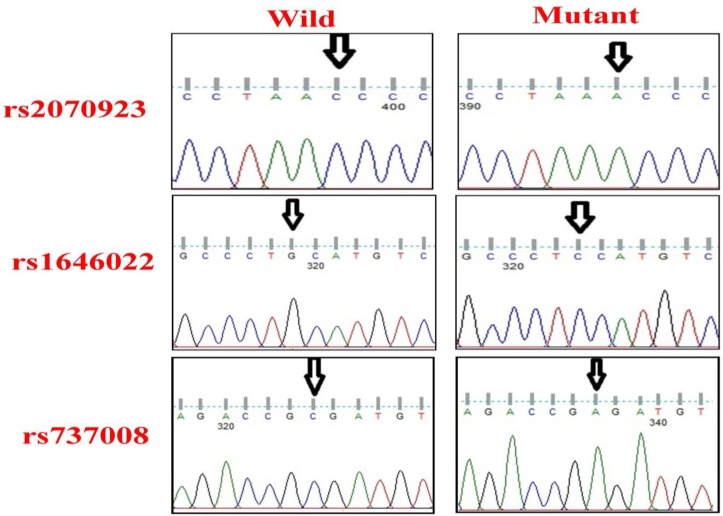
Chromatogram of DNA sequencing of *PRM1* and *PRM2* for detecting monoallelic expression.

Among these nine highly prevalent polymorphism areas (Table II), the allele frequencies of three SNPs in asthenozoospermia men including 373C>A (r s2070923) and 298G>C (rs1646022) in *PRM2* and 139C>A (rs737008) in *PRM1* were insignificantly higher than the control group (Figure 2).

**Table II:** The data of nine highly prevalent polymorphisms of *PRM1* and *PRM2*

**Gene**	**Allele change**	**RS**	**Global MAF**	**Functional consequence**	**Residue change**	**Amino acid position**
**PRM I**	102G>T AGG⇒AGT	rs35576928	A= 0.0026/13	Mmissense	R [Arg] ⇒ S [Ser]	34
	49C>T CGC⇒TGC	rs140477029	A= 0.0022/11	Missense	R [Arg] ⇒ C [Cys]	17
	139C>A* CGA⇒AGA	rs737008	T= 0.4878/2443	Synonymous codon	R [Arg] ⇒ R [Arg]	47
**PRM II**	248 C>T CAG⇒TAG	rs779337774	A= 0.000008/1	Stop gained	Q [Gln] ⇒ Ter[*]	50
	401 G>A CCG⇒CTG	rs545828790	A= 0.0008/4	Missense	P [Pro] ⇒ L [Leu]	134
	288 C>T CCC⇒CCT	rs115686767	A= 0.0355/178	Synonymous codon	P [Pro] ⇒ P [Pro]	96
	298G>C* GCA⇒CCA	rs1646022	G= 0.2708/1356	Missense	A [Ala] ⇒ P [Pro]	100
	373C>A*	rs2070923	T= 0.4491/2249	Intron variant		
	288G>C CCG⇒CCC	rs201933708	G= 0.0076/38	Synonymous codon	P [Pro] ⇒ P [Pro]	96

RS: Clustered RefSNPs

MAF: Minor allele frequency

PRM: Protamine

SNPs: Single nucleotide polymorphisms

* The symbol refers to the SNPs with different frequencies in asthenozoospermia and control groups.

**Table III:** Comparison on genotype and allele frequency of *PRM1* and *PRM2* gene polymorphisms between the asthenozoospermia and control groups

**Gene**	**SNP**	**Genotypes/ Alleles**	**Case group (n=92)**	**Control group (n=87) **	**p-value** *	**OR (CI)**
*PRM1*	rs737008	CC	33(47)	21(25)	Ref.	
		CA	47(40)	51(58)	0.124	0.489(0.160-1.492)
		AA	12(13)	15(17)	0.537	0.685(0.137-3.424)
		C	123(67)	93(53)	Ref.	
		A	61(33)	81(47)	0.135	0.721(0.473-1.099)
*PRM2 *	rs2070923	AA	26(28)	37(43)	Ref.	
		AC	52(57)	30(34)	0.164	2.576(0.839-7.907)
		CC	14(15)	20(23)	0.726	0.606(0.149-2.464)
		A	104(57)	104(60)	Ref.	
		C	80(43)	70(40)	0.592	1.141 (0.750-1.740)
	rs1646022	GG	29(31)	31(36)	Ref.	
		GC	54(59)	49(56)	1.000	1.000(0.333-3.005)
		CC	9(10)	7(8)	1.000	1.565(0.239-10.241)
		G	112(61)	111(64)	Ref.	
		C	72(39)	63(36)	0.586	1.133 (0.738-1.738)
	rs201933708	CC	92(100)	85(97)	Ref.	
		CG	-	2(3)	1.000	0.490(0.371-0.649)
		GG	-	-	-	-
		C	184(100)	172(99)	Ref.	
		G	-	2(1)	0.235	0.187 (0.008-3.926)
	rs115686767	CC	92(100)	85(97)	Ref.	
		CT	-	2(3)	1.000	0.490(0.371-0.649)
		TT	-	-	-	-
		C	184(100)	172(99)	Ref.	
		T	-	2(1)	0.235	0.187 (0.008-3.926)
	rs545828790	CC	92(100)	84(97)	Ref.	
		CT	-	3(3)	1.000	0.490(0.371-0.649)
		TT	-	-	-	-
		C	184(100)	171(98)	Ref.	
		T	-	3(2)	0.132	0.495 (0.006-2.591)

Data presented as n (%).

PRM: Protamine

RS: Clustered RefSNPs

SNPs: Single nucleotide polymorphisms

* Fisher's exact test

PCR: Polymerase chain reaction

PRM: Protamine

PRM: Protamine

## Discussion

In the present study, 3 SNPs in PRM1 and PRM2 were identified, although the allele frequency between case and control groups was not significant. Nine areas with high probability of polymorphism occurrence (Table I) were screened in search of potential mutations. Some of these nine polymorphisms have been previously reported in protamines I and II of infertile or azoospermia men, but not asthenozoospermia (6, 9, 12). 139C>A was synonymous codon substitution with residue change of [Arg to Arg], while, 298G>C and 373C>A were a nonsynonymous substitution. 298G>C resulted to residue change of [Ala to Pro] and 373C>A is the intron variant. Therefore, 373C>A causes translation destructive interference and alteration of protein structure; while, 139C>A is functionally missense and 373C>A is functionally an intron variant (NCBI database). Hence, this SNP may not cause tremendous changes in the protein structure. Also, none of the SNPs was in the primer sequences, because all of the DNA samples were amplified to nearly the same extent. Except for 373C>A, the other SNPs were at different loci of coding regions. All of the reported SNPs were previously registered for protamines of infertile or azoospermic, but not asthenozoospermic men (6, 12).

In 298 G>C transversion, alanine was converted to proline. Proline cannot act as a hydrogen bond donor; hence it is known as “alpha helix breaker” and “beta sheet breaker” in protein structure with a high tendency to locate at the beginning of the helix and plays a role in signal transduction (16). Jamali *et al* evaluated the association of genetic transversions and idiopathic oligozoospermia in a group of the Iranian population. They found 190C>A and 298G>C polymorphisms in PRM1 and PRM2 of oligozoospermia patients. However, their data revealed no significant risk factor between 298G>C and oligozoospermia (17). Also, Venkateshand colleagues found no significant association between 298G>C & 373C>A SNPs and DNA integrity with male infertility (18). It was recently reported that 298G>C and 373C>A are associated with sperm count (19). 

Intronic regions, such as c.373C>A of *PRM2* are a highly conserved region of eutherian mammals protamine, thus any nucleotide changes in these regions would probably have a significant role in destructive gene expression and protein function (18). 139C>A SNP, as a missense polymorphism, cannot result to protein structure deformation. Two other SNPs can alter the spatial structure of protein, such that sperm DNA packaging and integrity may affect spermatozoa production and function. Parallel to a previous study (20), the present study did not find any significant differences between the 298G>C and 373C>A monoallelic mutation frequency and asthenozoospermia. Imken and co-worker reported that 139C>A is identified in the 5′ region of the gene, but its allelic frequency was similar in fertile and infertile subjects (21). In another study, it was considered as a common polymorphism in PRM1, but its functionality as a synonymous codon caused no proven pathogenicity (22). Previous studies reported these SNPs in PRM1 and PRM2 of infertile men, but the majority found no significant differences between patients and the control group (6, 12).

A meta-analysis evaluated the potential association between male infertility and 6 common SNPs of PRM1 & PRM2 including rs35576928, rs737008, rs35262993, rs2301365, rs1646022, rs2070923. The results revealed that 190C>A  (rs2301365)  polymorphism was a risk factor for male infertility (12).

Also, it was reported that 190C>A is associated with teratozoospermia (23), abnormal sperm morphology and increased protamine P1/P2 ratio (9). While, rs1646022 and rs737008 polymorphisms enforce protective effect against male infertility in the Asian population. Conservation of the protamine structure is important in mammals (12). Any minor changes in the coding and non-coding areas of PRM1 and PRM2 genes may lead to significant abnormalities in their expression and male fertility problems. These SNPs were reported as a male infertility risk factor in previous studies, but in this meta-analysis no significant correlation was found (12). 

The explanation is that geographical issues, ethnicity, control source and a number of recruited patients are of importance in polymorphism studies videlicet ethnicity may contribute to the difference in male susceptibility to infertility. For example, Tüttelmann and colleagues reported a correlation between the risk of infertility and rs1646022. They reported that this polymorphism is associated with mild oligospermia (5). But a meta-analysis by Jiang and co-worker in 2015 showed different outcomes unlike the past results. They revealed that rs1646022 noticeably decreased the risk of male infertility in the Asian population, because this mutation leads to failure of some key enzymes for cutting and jointing of the PRM2 gene during the protamine translation process. However, the results from the Caucasians and other regions are different, suggesting that ethnicity is a factor which should be considered in susceptibility. Also worthy of note is the fact that genes expression, environmental factors and spermatogenesis disorder play a great role in male sterility (12).

## Conclusion

Our findings showed that the presence of some SNPs (373C>A, 298G>C and 139C>A) in protamine genes is probably associated to asthenozoospermia although when compared to normal men, no significant difference was found which demand more evaluations.

**Suggestion**

To precisely correlate these gene alterations to asthenozoospermia, with significant difference, a larger population of asthenozoospermia patients from various ethnicities should be recruited for wide-spread evaluations.
